# Flock sensitivity and specificity of pooled fecal qPCR and pooled serum ELISA for screening ovine paratuberculosis

**DOI:** 10.1371/journal.pone.0226246

**Published:** 2019-12-26

**Authors:** Yoann Mathevon, Gilles Foucras, Fabien Corbière

**Affiliations:** UMR INRA ENVT 1225 IHAP, Ecole Nationale Vétérinaire de Toulouse, Toulouse Cedex, France; University of Illinois, UNITED STATES

## Abstract

The aim of our study was to evaluate the flock sensitivity and specificity of fecal qPCR and serum ELISA using pooled samples for screening paratuberculosis in French sheep.

Using individual feces with low or high qPCR Ct values from ewes sampled in 14 infected flocks, a total of 555 pools of size 5, 10 and 20 were created by diluting individual materials in negative feces and analysed using a commercial IS900 qPCR kit. The relative performances of pooled serum ELISA analysis were evaluated based on the analysis of 181 different pools of size 5 and 10, composed of individual serum samples of various individual S/P values. Results showed that for pools of size 5, 10 or 20, individual fecal samples with low Ct values were invariably detected. Conversely fecal samples with high Ct values were associated with a lower detection rate in both pools of size 5 (87.0% to 90.0%), 10 (63.0% to 70.7%) and 20 (46.7% to 60.0%). After lowering the decision threshold to 25% and 15% for serum pools of size 5 and 10 respectively, the pooled serum ELISA relative sensitivity ranged between 62.2% and 100.0% depending on the composition of the pools.

Finally, a simulation study was carried out to evaluate the performances of 16 screening strategies at flock level, with varying pool size (5 to 20) and number (5 to 60). The use of pooled serum ELISA led to very false positive detection rate ranging between 37.6% and 91.8% in paratuberculosis free flocks and prevents its further use in that context. For infection prevalence ≤ 5%, the flock sensitivity based on pooled fecal qPCR ranged between 39.0% (5 pools of size 10) and 99.9% (300 sampled individuals, with pools of size 5,10 or20), and was always above 93% when the infection prevalence was greater or equal to 15%. We conclude that pooled-fecal qPCR but not pooled-serum ELISA could be a useful tool to detect sheep flocks infected with paratuberculosis.

## Introduction

Paratuberculosis is a chronic infectious disease affecting the digestive tract of ruminants, caused by *Mycobacterium avium* subsp. *paratuberculosis* (*Map*). Correct classification and estimation of the prevalence in infected herds/flocks are important to provide guidance for control programs, including vaccination and/or restrictions on animal movements. Flock or herd-testing strategies based on individual milk or serum ELISA and fecal culture have been assessed in several studies but appear expensive [1–3]. Furthermore, the lack of sensitivity of individual tests, especially for the detection of sub-clinically infected animals [4,5], leads to inaccurate negative predictive value regarding the individual infectious status. In sheep production, the main objective of paratuberculosis surveillance programs is usually to prove freedom of disease or to estimate prevalence rather than to identify individual infectious animals.

Pooled sample testing (PST), proposed initially for screening syphilis in US soldiers during World War II [6], has become increasingly popular to monitor paratuberculosis in herds/flocks as it allows substantial savings in laboratory costs [7,8]. For detection of *Map*, PST was originally applied to faecal culture by physically combining and mixing a number of individual fecal samples [7,9,10] and has been extended to polymerase chain reaction (qPCR) testing [11,12]. Bulk tank milk ELISA [13] or qPCR [14,15] and environmental sampling, including manure pit [16], have also been proposed, mostly in dairy cattle, and can be viewed as extreme pooling strategies [17].

However, whatever the approach, combining and mixing individual samples may lead to a drop in the concentration of *Map* or antibodies toward *Map* to a level that cannot be detected [18], making the sensitivity of the pooled-sample approach lower than the approach based on individual testing. Therefore, when evaluating the diagnostic accuracy of pooled-sample based herd/flock-testing, the influence of the dilution effect on pool sensitivity is an essential prerequisite. Furthermore, depending on the surveillance purposes, the best testing strategy (i.e. number and pool size per herd/flock) may differ and should be evaluated.

The analytical sensitivity of pooled-sample approach based on *Map* detection may vary according to sample quality, pooling and mixing methods, culture media [19], DNA extraction methods, DNA target and qPCR systems [20–22]. Similarly, the accuracy of bulk tank milk antibody detection may depend on the ELISA kit used or to the decision threshold applied [13,23]. Finally, it is unwise to simply extrapolate already published estimates to any other method. Most of all, the intensity level of individual samples composing the pool (i.e. number of *Map* or antibody titers) may have a strong influence on the pool result [9,24]. To our knowledge, the detection of antibody response toward *Map* based on pooled serum samples has not been published yet. This approach has however already been evaluated for other sheep or porcine diseases [25,26] and showed that decision thresholds should be re-evaluated for different pool sizes to allow a satisfactory detection rate.

In this context, we aimed at evaluating the flock sensitivity and specificity of pooled fecal qPCR and pooled serum ELISA for screening ovine paratuberculosis. In an experimental study, we first evaluated the effect of dilution on the sensitivity and specificity of pooled fecal or serum testing. Based on this experiment and on the distribution of individual results in commercial infected and non-infected flocks, we conducted a simulation study in order to evaluate the sensitivity and specificity of various screening strategies at the flock level. Finally, we looked at whether a crude estimation of the true within flock prevalence of infection could be achieved for these different screening strategies.

## Material and methods

### Flocks, animals and biological samples

The diagnostic test results from a cross-sectional study were previously published by the study investigators [27]. Briefly, 1197 individual serum and fecal samples were collected from 2- to 3-year-old sheep without clinical signs of paratuberculosis in 14 purebred Causse du Lot closed meat sheep flocks where paratuberculosis infection was endemic. Fecal excretion was determined using qPCR based on IS900 sequence detection, and serology was performed using a commercial ELISA. Beyond the binary (positive/negative) test results that were analysed in this study, we also evaluated the distribution of the quantitative responses for serum ELISA (i.e sample to positive ratio, S/P) and fecal qPCR (i.e. cycle threshold, Ct) across the sampled population.

Blood and fecal samples were also collected from 387 ewes in three flocks known to be free from paratuberculosis (closed purebred Lacaune flocks) using the same sampling methods.

Blood and fecal samplings occurred as part of routine veterinary examination within the local voluntary paratuberculosis surveillance plan and were performed by accredited veterinary practitioners. This study was carried out in strict accordance with the recommendations established by the European Commission Directive 2010/63/UE. All animals used in this study were handled in strict accordance with good clinical practices and all efforts were made to minimize suffering. All animal owners gave written consent for their animals’biological samples to be used in this study.

### Pooled-sample analysis

We hypothesized that pooled-sample testing was sensitive to the quantity of *Map* DNA or of antibodies against *Map* presents in individual fecal / serum samples that composed the pool, i.e. that the effect of dilution was less detrimental for pooled-sample sensitivity when samples were strongly test-positive at the individual level. For practical reasons, only a limited number of serum and fecal samples could be included in the experiment, and simplifications were made in order to assess the effect of dilution. The continuous test results were therefore discretized in different ordered response categories. Note that for both serum ELISA and fecal qPCR, these response categories did not rely on established biological knowledge but were rather driven by practical considerations related to the pooled-sample analysis and the design of the simulation study.

For serum ELISA the decision threshold of S/P>45% and S/P≥55% are defined by the manufacturer to distinguish between negative, doubtful and positive results, respectively. Based on these cut-off values, individual S/P results were arbitrarily separated in four response categories as follows: S/P < 22.5%: negative low (LN); [22.5% - 45.0%]: negative high (NH); [45.0% - 90.0%]: positive low (PL) and S/P ≥90.0%: positive high (PH).

For fecal qPCR, two response categories were used, based on the distribution of Ct values of the samples recovered in the 14 investigated flocks infected with paratuberculosis (see below). We arbitrarily classified samples with Ct < 30 as “highly contaminated” (HC) with *Map* and those with the Ct ≥ 30 as “lowly contaminated” (LC).

### Pooled serum ELISA

Pools of size 5 and 10 were manually constituted in duplicate using 20μl of each individual serum sample. Samples with individual S/P value below 22.5% were used as negative diluent.

To evaluate the ability of pooled serum ELISA to correctly retrieve pools containing at least one individual ELISA positive sample, pools containing either one "positive high (PH)", 1 "positive low (PL)" and 2 "positive low" individual sample(s) were investigated. Conversely, to evaluate whether pooled serum ELISA could yield false positive results, we constructed pools containing either only "negative low (NL)" and 1 up to 3 "negative high (NH)" individual sample(s). Overall, using 13 PH, 37 PL, 41 NH, and 156 NL unique individual samples, 181 pools were created for each pool size.

### Pooled fecal qPCR

Feces from 6 individual ewes with Ct < 30 (range 20.42–28.12) and 9 with Ct ≥ 30 (range 34.8–41.7) from the investigated flocks were used to build the pools. Confirmed qPCR-negative fecal samples collected from uninfected animals in a research flock known to be free of paratuberculosis (closed purebred Lacaune flock) were used for creation of pools. Before pooling, positive feces from each individual ewe were mixed thoroughly. Three grams of each positive fecal sample were mixed manually in a sterile plastic bag until complete homogenization with either 12, 27 or 57 grams of negative feces. The same was also done using 10 grams of positive feces and either 40, 90 or 190 grams of negative diluent. Thus, the pools contained positive and negative feces in a ratio of 1:4, 1:9 or 1:19 which is equivalent to creating pools of size 5, 10 and 20. A total of 195, 210 and 150 pools of size 5, 10 and 20, respectively, were created to evaluate the relative sensitivity of pooled fecal qPCR (PRSe_qPCR_). In addition, 40 pools containing only negative feces were constructed to evaluate the specificity of pooled fecal PCR (PRSp_qPCR_).

### Laboratory testing

A commercial serum ELISA (IDEXX paratuberculosis screening kit, batch 5074, IDEXX, Montpellier, France) was applied to individual or pooled serum samples using an overnight incubation protocol following the manufacturer’s instructions, as previoulys described [27].

Individual and pooled fecal samples were submitted to a commercial qPCR protocol targeting the IS900 DNA fragment of Map (Adiafilter and Adiavet ParaTB Real Time, BioX, Rochefort, Belgium) using 10 grams as working material, as previously described [27].

#### Relative accuracy of pooled-sample analysis

The relative sensitivity of pooled serum ELISA (PRSe_ELISA_) and pooled fecal qPCR (PRSe_qPCR_) were defined as the probability of pools containing at least one test-positive individual sample that yielded a positive result, i.e. Pr(pool test +| ≥ 1 test positive individual sample). The pool relative specificities PRSp_ELISA_ and PRSp_qPCR_ were defined as the proportion of pools containing only test-negative individual samples that yielded a negative result, i.e. Pr(pool test −| only test negative individual samples).

For serum ELISA, when determining whether pools are test positive or negative, it can be beneficial to establish alternative cut-offs that are lower than those recommended by the manufacturer. Indeed, pooling method dilutes test-positive individual samples, causing a lower signal that requires a lower cut-off to be regarded as positive. However, choosing very low cut-offs may lead to false positive results in true negative pools, especially when several NH samples are pooled together. Therefore, PRSe_ELISA_ and PRSp_ELISA_ estimates were computed and compared for different decision thresholds ranging from S/P>10% to S/P>45%.

For our experiment, we used individual qPCR positive fecal samples coming from a few number of independent ewes (n = 15) to build numerous fecal pools. These pools may therefore be highly correlated. To check whether the ewe effect was influencing, mixed logistic regression models were fitted with an ewe random term that allowed accounting for the clustering of pools at the ewe level. The response variable was the pool test result (positive/negative). The amount of indivivual feces used to build the pool (3 or 10 grams), the pool size (5, 10 or 20) and the interaction between the two variables were treated as fixed effects. Separate models were fitted for pools built with HC and LC samples.

#### Flock level sensitivity and specificity of pooled-sample testing

An individual-based simulation study was carried out to estimate the flock-sensitivity (FPSe) and flock-specificity (FPSp) of pooled fecal qPCR and pooled serum ELISA in sheep in simulated flocks with a range of infection prevalence levels and under different sampling and pooling scenarios. We also looked at whether a crude estimation of the true within flock prevalence of infection could be achieved for these different screening strategies. For ease, some results from the experimental study were merged when they were similar.

Paratuberculosis infected and uninfected flocks with 300 sheep were simulated with infection prevalence ranging from 0% (paratuberculosis free flocks) to 30%. The number of ewes sampled in each flock was 50 or 100. The sizes of 50 corresponds to the minimal number of sheep to sample and test each year in France for the national mandatory brucellosis surveillance plan. We also investigated scenarios where all animals within flocks were sampled (i.e n = 300) and submitted to pooled serum ELISA or pooled fecal qPCR. Although this situation is unlikely to occur in France due to logistical and cost constraints, it allowed us in estimating the lowest infection prevalence that could be detected based on pooled-sample analysis for this flock size.

The simulation program comprised 3 independent compartments, hereafter named the flock, individual and pool compartments.

In the flock compartment the number of truly infected and truly uninfected sheep within a flock was determined, given the flock size and the true infection prevalence. For each animal, the binary (positive/negative) status of its serum or fecal sample was then modelled in the individual compartment, given the sensitivity (ISe_ELISA_, ISe_qPCR_) and specificity (ISp_ELISA_, ISp_qPCR_) of diagnostic tests applied at the individual level. For infected flocks, these estimates were derived from Mathevon *et al*. (2017) [27] in which the diagnostic accuracy of the IDEXX serum ELISA and the ADIAVET fecal qPCR were estimated in sub-clinically infected sheep using Bayesian modeling. Interested readers are referred to Mathevon *et al*. (2017) [27] for further details about the estimating procedure. For paratuberculosis-free flocks, the specificity of serum ELISA test at the individual level was computed based on results obtained in the present study. In contrast to serum ELISA, fecal qPCR at the individual level was assumed to be perfect in paratuberculosis-free flocks. Given individual binary test results, each individual was then allocated to a serum ELISA (negative, PL, PH) and fecal qPCR (LC, HC) response category, based on the probability distribution functions observed in the 14 flocks infected and the 3 paratuberculosis-free flocks investigated in the present study.

Finally, random sampling of individual sheep, random creation of pools and pool analysis were modelled in the pool compartment of the simulation program. For a given fecal pool (respectively serum pool), the probability that it yielded a positive qPCR (respectively ELISA) result was modelled based on the relative diagnostic performances which were estimated in the experimental part of our study.

For each combination of infection prevalence, number and size of pools, 1000 flocks were simulated, each being sampled over 1000 iterations. For each iteration, the number of pools that yielded a positive test result was recorded for each simulated flock and a flock was considered to be positive when a single pool was found positive. Flock sensitivities of pooled serum ELISA (FPSe_ELISA_) and of pooled fecal qPCR (FPSe_qPCR_) were computed as the proportion of infected flocks that yielded at least one positive pool result and flock specificities (FPSp_ELISA_ and FPSp_qPCR_) as the proportion of paratuberculosis-free flocks that yielded no positive pool result at all. Finally, the overall FPSe_ELISA_, FPSe_qPCR_, FPSp_ELISA_ and FPSp_qPCR_, number of positive pools and 95% confidence interval were calculated using the median value and the 2.5% and 97.5% percentiles of the estimate distributions over the 1000 iterations.

**Fig 1:**
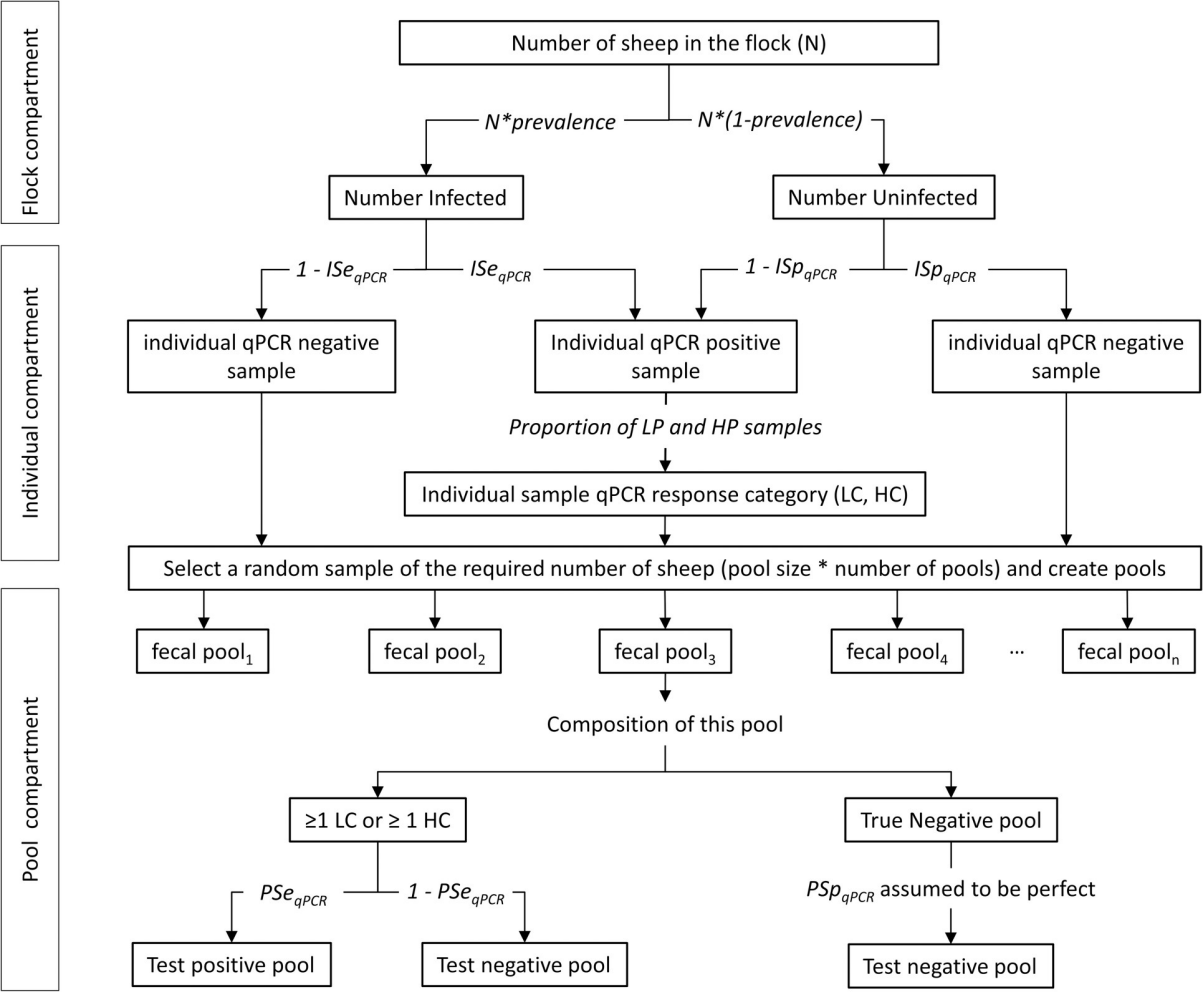
Schematic representation of the simulation study approach for pooled-fecal qPCR. ISe_qPCR_: individual sensitivity; ISp_qPCR_: individual specificity; PRSe_qPCR_: pooled-sample relative sensitivity for fecal qPCR; PRSp_qPCR:_ pooled-sample relative specificity for fecal qPCR; LC sample: Low Contaminated (Ct ≥ 30) fecal sample; HC sample: Highly Contaminated (Ct < 30) fecal sample.

The schematic representation of the simulated sampling approach is summarised in Fig 1 for fecal qPCR and is provided as additional file S1 Fig for serum ELISA. Details on input parameters are also provided in additional file S2 Table.

#### Statistics

Data analysis and simulations were performed using the R software [28]. Frequencies and detection rates were compared using the Fisher exact test. Mixed logistic regression models were fitted using the glmer function from the lm4 package [29]. Simulations were performed using package “simulator” [30].

## Results

### Individual test result distributions

**Fig 2:**
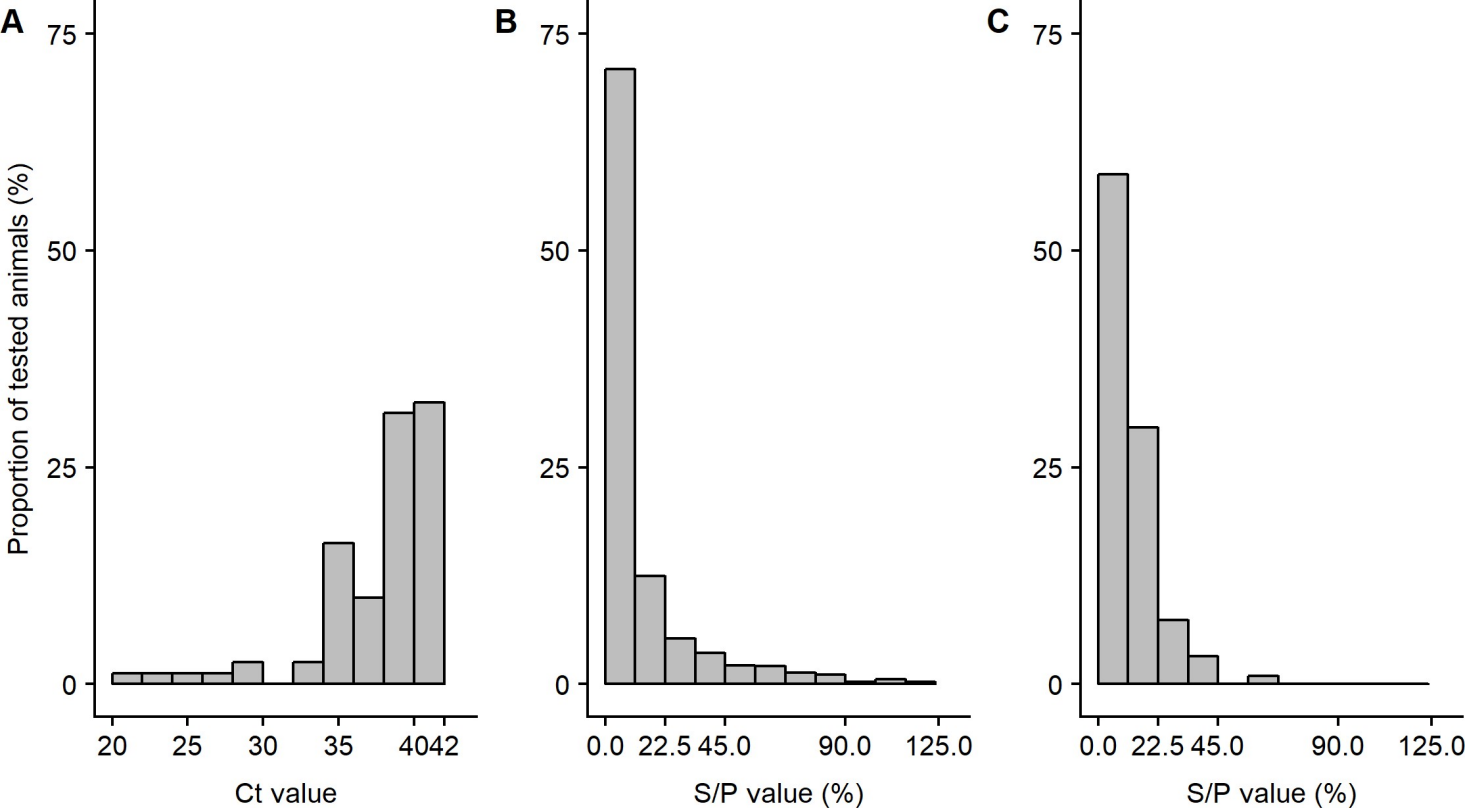
Distribution of individual serum ELISA S/P values and fecal qPCR Ct results in 14 paratuberculosis infected and 2 paratuberculosis-free sheep flocks. A: fecal qPCR Ct in infected flocks; B: ELISA S/P values in infected flocks; C: ELISA S/P values in paratuberculosis-free flocks.

The distributions of quantitative results for individual serum ELISA (S/P values) and fecal qPCR (Ct) are shown in Fig 2 for the 14 infected and the 3 paratuberculosis-free flocks. More detailed results are provided at the flock level in S2 Table.

#### Individual serum ELISA results

Overall, at the S/P > 45% decision threshold, 93 individual samples were considered as positive in infected flocks, with 13 samples (14.0%) classified as “Positive High” (PH, S/P value ≥ 90%). At the flock level, the ELISA-based individual apparent prevalence ranged between 1.1% and 16.5% (median value 7.2%). PH serum samples were found in only 7 out of 14 infected flocks. Among the 1104 negative results, 106 (9.6%) had S/P values between 22.5% and 45.0% and were regarded as “Negative High” (NH). The proportion of NH samples amongst negative one differed between flocks (range 3.1–26.8%). Finally, over the 211 individual serum ELISA results available in paratuberculosis-free flocks, 2 “Positive Low” (PL) samples were found in two different flocks while 23 (10.7%) negative results had NH values.

#### Individual fecal qPCR results

No positive result was found in the 387 ewes sampled from the three paratuberculosis free flocks. In infected flocks 105 samples (8.78%) yielded a positive qPCR response, with intra-flock individual apparent prevalence ranging from 0.0% to 29.1% (first quartile 3.0%, third quartile 12.8%). Among those samples, only 6 (5.7%) with Ct < 30 were found in 5 different flocks and were classified as “Highly Contaminated” (HC) (Fig 2).

### Relative performances of pooled-sample analysis

#### Pooled serum ELISA

As expected, pooled-serum ELISA was strongly influenced by both pool size and pool composition. Results are summarized in Tables 1 and 2 as numbers of pools that yielded a positive response at different chosen S/P decision thresholds for pools of size 5 and 10, respectively. Retaining the S/P>45% threshold that is applied to individual serum samples led to poor detection rates, especially when only one PL serum sample was incorporated (21.6% and 0% for pool size 5 and 10, respectively). Lowering the decision threshold improved the detection rate, but choosing very low cut-off values led to false positive results. For pools of size 5, the S/P>25% threshold was associated with only negative results for all investigated combinations of NL or NH individual samples (PRSp_ELISA_ = 100.0%) and results were therefore merged in a “negative” group for the simulation study.

**Table 1:** Pooled-serum ELISA results for pools of size 5.

Pool composition*	# tested	# positive for different S/P (%) decision thresholds				
		>20%	>25%	>30%	>35%	>45%
5 NL	30	0	0	0	0	0
1 NH	41	2	0	0	0	0
2 NH	21	4	0	0	0	0
3 NH	10	2	0	0	0	0
1 PL	37	28	23	21	19	8
2 PL	21	21	21	21	20	19
1 PH	21	21	21	20	20	18

* pool composed with serum samples of various S/P values at the individual level. NL: Negative Low: S/P < 22.5%; NH: Negative High: 22.5% ≤ S/P ≤ 45.0%; PL: Positive Low: 45.0% < S/P < 90.0% and PH: Positive High: S/P ≥ 90.0%.

Number of pools that were deemed to be positive given the S/P decision threshold.

**Table 2:** Pooled-serum ELISA results for pools of size 10.

Pool composition*	# tested	# positive for different S/P (%) decision thresholds				
		>10%	>15%	>20%	>25%	>45%
10 NL	21	1	0	0	0	0
1 NH	38	7	0	0	0	0
2 NH	27	5	0	0	0	0
3 NH	16	3	0	0	0	0
1 PL	37	32	24	17	14	0
2 PL	21	21	21	20	20	8
1 PH	21	21	21	19	19	11

* pool composed with serum samples of various S/P values at the individual level. NL: Negative Low: S/P < 22.5%; NH: Negative High: 22.5% ≤ S/P ≤ 45.0%; PL: Positive Low: 45.0% < S/P < 90.0% and PH: Positive High: S/P ≥ 90.0%.

Number of pools that were deemed to be positive given the S/P decision threshold.

This decision threshold allowed for moderate to high detection rates (PRSe_ELISA_ = 62.2% for pools containing one PL sample and PRSe_ELISA_ = 100% for pools with 2 PL samples or 1 PH sample). For pools of size 10, almost identical performances were obtained when applying the S/P>15% decision threshold (Table 2). The performances associated to these decision thresholds were therefore selected for the simulation study and were hereafter assumed to be equal for both pool sizes.

#### Pooled fecal qPCR

None of the 40 truly-negative fecal pools yielded a positive qPCR result, indicating that the analytical specificity of pooled fecal qPCR was perfect (PRSp_qPCR_ = 100%).

The mixed logistic regression models fitted to pools containing one Highly Contaminated (HC) sample did not converge because all pools except one gave a positive qPCR result (see below). When fitted to pools built with one Lowly Contaminated (LC) sample, the ewe random effect was not significant (based on the likelihood ratio test), and the PRSe_qPCR_ estimates were only marginally modified (results not shown). So for simplicity and ease of comprehension, the crude PRSe_qPCR_ estimates, assuming no ewe effect, are presented.

All except one of the 367 pools constructed by including a HC sample were detected by qPCR, whatever the pool size (5, 10 or 20) or the amount of feces (3 or 10 grams) used to construct the pools (Table 3). Conversely, 20/23 (87.0%) and 45/50 (90.0%) pools of size 5 containing one LC sample and constructed with 3 and 10 grams of positive feces were detected, respectively. Pools of size 10 and 20 containing one LC sample were even less frequently detected, with 46.7% to 70.7% of positive results (Table 3). For pools containing one LC sample, increasing the pool size from 5 to 10 or 20 lead to a significant decrease in detection rate (p = 0.0019 and p = 0.0001, respectively), which was not the case for pools containing one HC sample (p = 0.4939 and p = 1, respectively). The detection rates between pools of size 10 and 20 did not differ significantly (p = 0.1839). Finally, it is noteworthy that detection rates were not statistically different (Fisher exact test, all p > 0.05) whatever the individual fecal amount (3 or 10 grams) used to construct the pools, indicating that pooling higher amounts of feces did not lead to substantial improvement of sensitivity. Results obtained whith 3 and 10 grams were therefore merged for the simulation study.

**Table 3:** Pooled-fecal qPCR results for the detection of *Map* DNA.

Pool composition*	Individual fecal amount (g)	Pool size	# positive / # tested	% positive
1 HC	3	5	62 / 62	100.0
		10	65 / 65	100.0
		20	60 / 60	100.0
	10	5	59 / 60	98.3
		10	60 / 60	100.0
		20	60 / 60	100.0
				
1 LC	3	5	20 / 23	87.0
		10	17 / 27	63.0
		20	9 / 15	60.0
	10	5	45 / 50	90.0
		10	41 / 58	70.7
		20	7 / 15	46.7

*HC: Highly Contaminated fecal sample, qPCR Ct < 30; LC: Lowly Contaminated fecal sample, qPCR Ct ≥ 30.

#### Flock level sensitivity and specificity of pooled-sample testing

**Fig 3:**
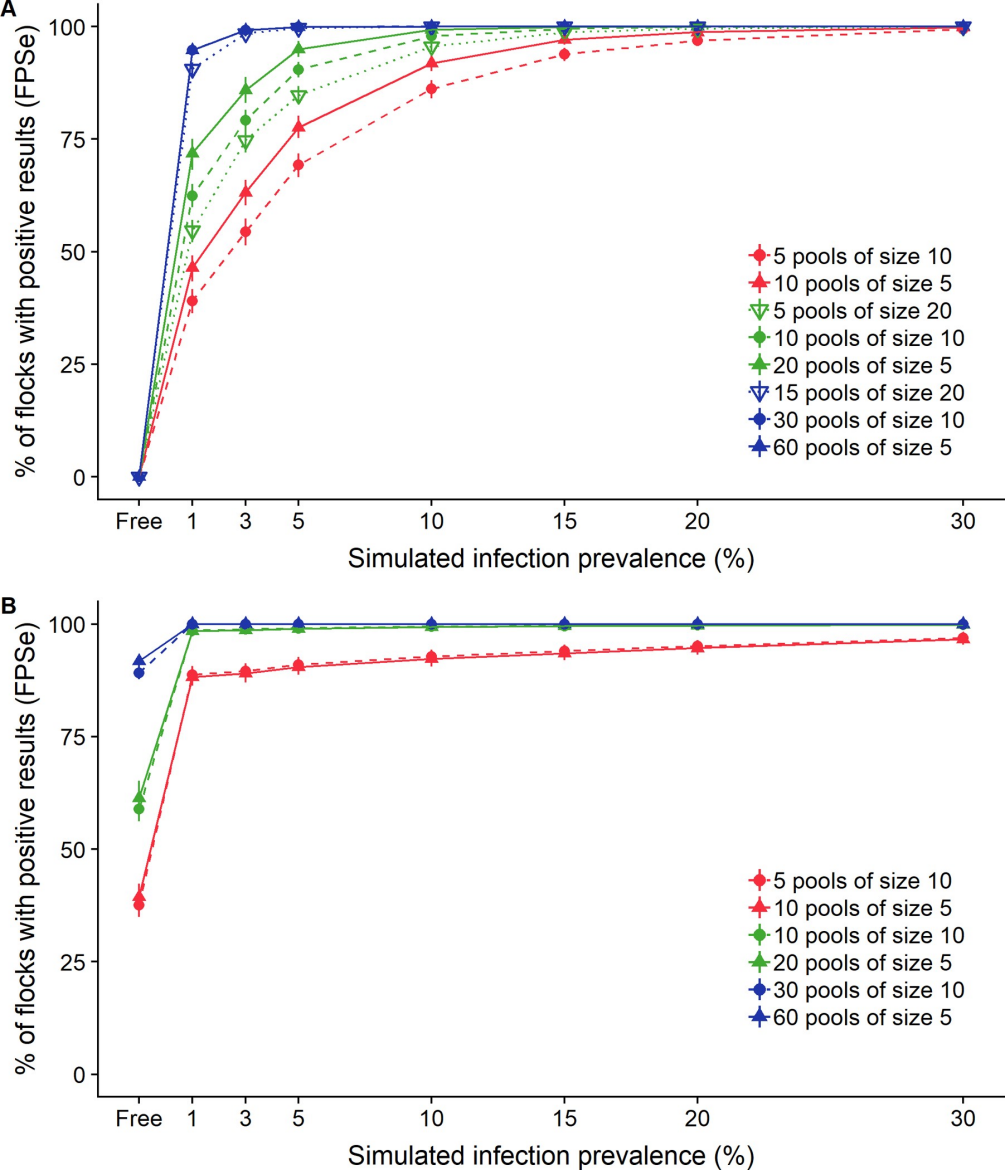
Proportion of flocks with at least one test-positive pool, according to number and size of tested pools and simulated infection prevalence. A (top): screening strategies based on pooled-fecal qPCR; B (bottom): screening strategies based on pooled-serum ELISA. Red: 50 sampled animals; green: 100 sampled animals; blue: 300 sampled animals.

The proportion of flocks that yield at least one positive pool result given the infection prevalence is shown in Fig 3 for different screening strategies. Not surprisingly, the flock sensitivity of pooled serum or pooled fecal screening strategies increased with the within-herd prevalence of infection and the number of animals sampled. Whatever the sampling strategy, very high detection sensitivities (> 93.0%) were found for both pooled-serum ELISA and pooled-fecal qPCR for infection prevalence greater or equal to 15%. For low infection prevalence (≤ 5%), the flock sensitivity of pooled-sample serum ELISA (FPSe_ELISA_) was still high (point estimates from 88.2% to 100.0%) and greater than the one associated with pooled-sample fecal qPCR (FPSe_qPCR_ between 39.0% and 99.9%). These very high detection rates for serum ELISA based screening strategies were however partially due to a lack in specificity, as shown by false positive detection rate in paratuberculosis-free flocks, ranging from 37.6% (5 pools of 10 samples) to 91.8% (60 pools of 5 samples). By construction, assuming a perfect specificity of fecal qPCR at the individual and pool level in paratuberculosis free flocks led to no false positive result.

For a given number of sampled animals (i.e. 50, 100 or 300), pooled-serum ELISA yielded virtually the same results whatever the pool size (5 or 10). For pooled-fecal qPCR, the same conclusion was drawn when infection prevalence was above 10% and/or when all animals within the flock were sampled. Conversely, when infection prevalence was below 10% and only 50 or 100 animals were sampled, testing small pools was associated with a better flock sensitivity (difference range for FPSeq_PCR_: 4.5% to 17.2%).

#### Estimation of within flock infection prevalence using pooled-sample testing

**Fig 4:**
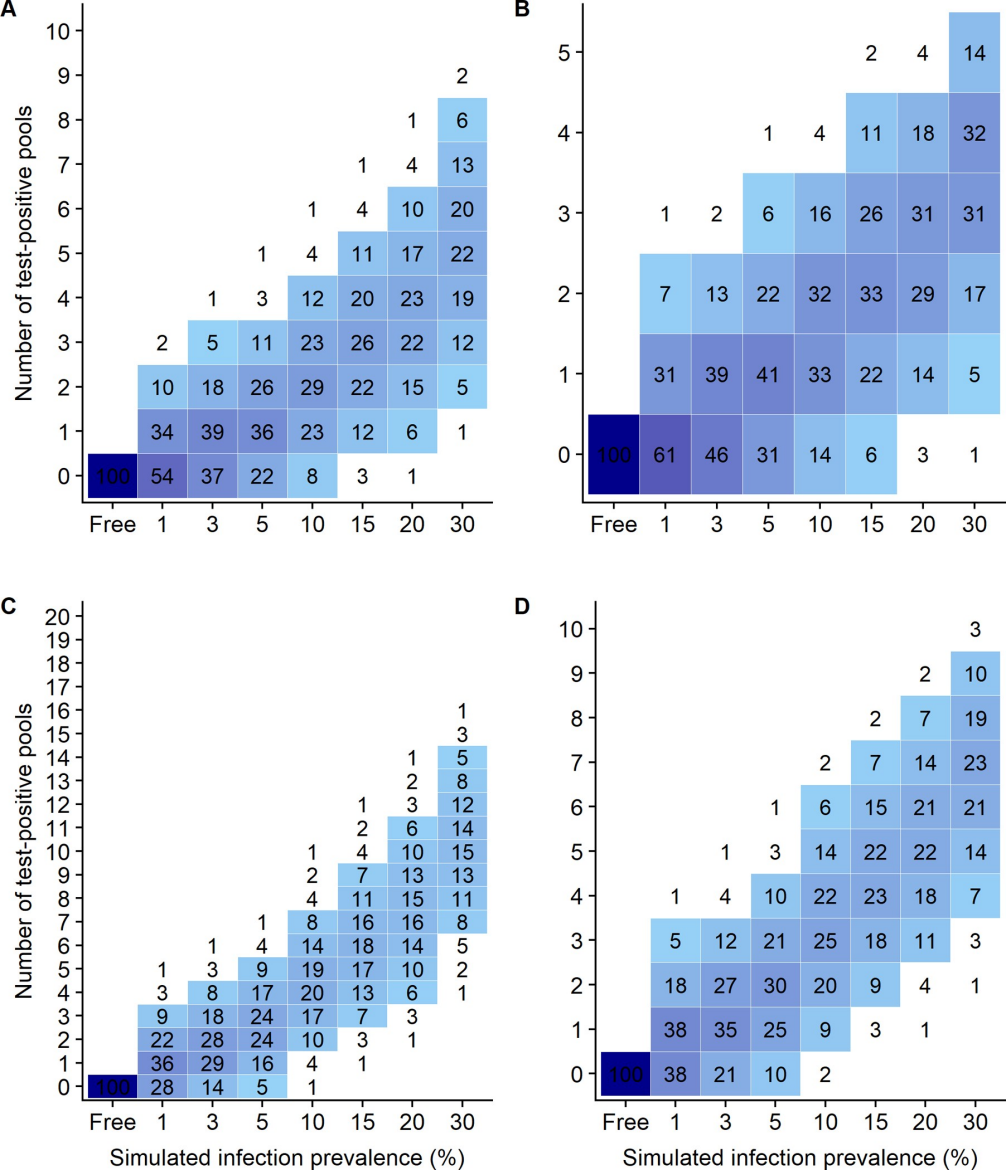
Number of qPCR-positive fecal pools detected, according to number and size of tested pools and simulated infection prevalence. A: 10 pools of size 5; B: 5 pools of size 10; C: 20 pools of size 5; D: 10 pools of size 10. For a given simulated infection prevalence, figures in cells give the mean proportion of flocks that yield a given number of qPCR-positive fecal pools.

The number of test-positive pools was also recorded for each screening strategy. Results are shown in Fig 4 for pooled fecal qPCR (pools of size 5 and 10 up to 100 sampled sheep). Results for other screening strategies (i.e. 5 pools of size 20, 15 pools of size 20, 30 pools of size 10 or 60 pools of size 5) are provided in S2 and S3 Figs for pooled-fecal qPCR. Clearly screening strategies based on the analysis of 50 animals were not informative enough to distinguish between the different simulated intra-flock infection prevalences. For instance, 1 fecal qPCR test-positive over 5 pools of 10 samples could be obtained for infection prevalence of 1% to 20% with probability ranging from 14% to 41% (Fig 4 panel B). Increasing the number of sampled animals (10 pools of 10 samples, Fig 4 panel D) led to slightly more precise estimation of the within flock infection prevalence. Under this screening strategy, getting no test-positive result only ensured with a high confidence level that the true infection prevalence is less than 10%, while when 5 test-positive pools over 10 were observed, the infection prevalence was likely to be higher than 5%. Finally testing 30 pools of 10 fecal samples or 60 pools of 5 fecal samples (S2 and S3 Figs) was expectedly associated with the more precised within-flock infection prevalence estimates. In these settings, getting no or only one test-positive pool associated with a within-flock infection prevalence lower or equal to 3% in most cases.

## Discussion

The main objective of our study was to evaluate the performances of screening strategies based on serum or fecal pooled samples for detection of sheep flocks infected with paratuberculosis. In the absence of available estimates from the literature, it was appropriate to perform a laboratory evaluation of pooled-sample analysis for both serum ELISA serology and fecal qCPR.

Our estimates of the pool relative sensitivity (PRSe) and specificity (PRSp) are different from the diagnostic pool sensitivity (PSe) and specificity (PSp) defined by Christensen and Gardner (2000) [31] as PSe = Pr(pool test + | pool I+) and PSp = Pr(pool test −| pool I−) respectively. In the foregoing, “pool I+” means that a pool contains at least one individual sample collected from a truly infected animal, and “pool I–” means that a pool contains individual samples coming only from uninfected animals. In the meaning of these authors, PSe and PSp refer to the ability of pooled-sample testing to correctly detect individual animals of a given infectious status (infected, uninfected) [5] within a pool. In fact, due to the long and complex physiopathology of the disease, some infected or infectious individuals may not shed a detectable amount of *Map* in their feces or have mounted a detectable antibody response toward *Map* at the time of sampling. Conversely some truly uninfected animals may yield positive individual test results, for instance due to an imperfect analytical specificity or because the presence of passively shed MAP in their feces [32,33]. Our estimates PRSe and PRSp only refer to the ability of pooled-sample analysis to correctly classify pools containing individually tested positive or negative samples (whatever the true status of the animal they are coming from). In other words, we evaluated the probability of positive and negative agreement between individual and pooled-sample testing for different pool compositions and sizes.

Our assumption was confirmed that the individual level of response had an influence on the pool detection rate for both ELISA and fecal qPCR. Indeed, the detection was close or equal to 100% for pools containing a strongly positive serum or fecal sample, while it was lower, but still acceptable (between 46.7% and 89.0%), when only one low positive sample was included. The detection sensitivity for qPCR applied to 5 to 20-feces pools containing a low positive sample (Ct ≥ 30) was similar or greater than the one obtained for pooled fecal culture (PFC) using BACTEC 12B or 7H10 media in sheep with the paucibacillary form of paratuberculosis (sensitivity between 15% and 60%) [9,24] or Herrold’s egg yolk (HEY) agar of liquid media in low shedder cattle (sensitivity between 20% and 63%) [34,35]. However, comparison between these PFC relative sensitivity estimates and those obtained for pooled fecal qPCR is difficult because fecal culture was not performed in our study and the classification of our fecal samples as originating from paucibacillary/low shedder or multibacillary/ high shedder sheep was therefore not possible. In accordance to other studies [12] our results however show that pooled fecal qPCR might be a useful alternative to fecal culture, because of its rapid turnaround time for test results and lower susceptibility to sample freezing. Note, however, that the extrapolation of our results to other qPCR methods should be done with caution, as performances of sample preparation, DNA extraction and amplification steps may highly vary between qPCR kits [22,36]. In particular, the use of a microfiltration and concentration step for fecal samples preparation and the use of magnetic bead isolation before DNA extraction might may be recommended to enhance detection sensitivity [37,38]. One other practical issue that may be encountered is the quality of fecal homogenate than is submitted to culture or qPCR analysis. *Map* is known to aggregate in clusters within feces [39] and, for stochastic reasons, this lack of homogeneity of distribution could explain the lower detection rate generally observed with lowly contaminated fecal samples from both cattle and sheep [9,24,34,35]. Moreover, compared to cattle, sheep feces are excreted in dry and firm pellets that are difficult to manually break apart and homogenize. Several approaches could be used to tackle this issue, including the use of a stomacher system with added saline [40] or applying pooling methods that allow for higher feces volumes submitted to DNA extraction [20]. In our experiments we did not find any difference between pools built with 3 or 10 grams of individuals fecal samples, indicating that pooling 3 grams of individual feces, which is easier from a practical point of view, would be efficient enough.

To evaluate the relative performances of pooled serum ELISA, we lowered the cut-off value to compensate the dilution of positive samples. This approach has already been proposed for other disease in ruminants [25,41] or pigs [26] to improve sensitivity. As the lack of specificity is the main concern when screening a large population for a low-prevalence disease, we redefined the S/P decision threshold to maximise the relative sensitivity of pooled serum ELISA while keeping the pool relative specificity to 100%. This increase in sensitivity was mainly observed for pools containing only one lowly positive (PL) individual sample. Using the newly defined decision thresholds, the relative performances of pooled serum ELISA were comparable to those obtained for pooled fecal qPCR and satisfactory enough from an epidemiological point of view. As for qPCR, the extrapolation of our results to other ELISA commercial kits should be done with caution.

The simulation study carried out allowed us to evaluate the performances of different screening strategies based on pooled-sample testing, varying according to the number and size of tested pools. Unlike Dhand et al. [24] who evaluated the sensitivity of pooled faecal culture to detect Map excretion at the flock level, our target condition was animals infected with Map. Indeed, samples entering the pools may come from infected sheep that have not yet mounted a detectable antibody response towards Map or that do not shed enough bacteria in their feces to potentially test positive on fecal qPCR at the timing of sampling. Thus, the true infection prevalence is always higher than the apparent shedding prevalence or seroprevalence and the probability that an individual fecal or serum sample (that may be incorporated in a pool) yields a positive or negative test result needed to be estimated. To this end, the parameter estimates for sensitivity were those evaluated in a previous study performed in the same infected flocks [27]. Conversely, the input parameters used for the serum ELISA and fecal qPCR specificity at the individual level, were different between infected and uninfected flocks. Although this approach may appear unusual, it relies on several biological and epidemiological arguments. Indeed, while the analytical specificity of qPCR was considered to be perfect in free flocks based on results from Plain *et al*. [38], its epidemiological specificity was estimated to be 99.0% [97.6–99.6] in infected flocks [27]. This lack of perfect specificity regarding the actually true infected status of animals might reflect the potential of pass through of orally ingested organisms by uninfected individuals in infected flocks [32,33]. Similarly, some animals exposed to Map could develop a detectable serological response, without becoming permanently infected [42]. For both fecal qPCR or for ELISA serology, these "false-positive" results reveal an actual exposure to Map and the infection of the flock. In addition, the analytical specificity of the ELISA serology is known not to be perfect because of possible cross-reactions with other environmental Mycobacteria [43–45], even if an absorption step with *M*. *phlei* is now commonly incorporated in commercial serum ELISA kits. This is also reinforced by the observation of 2 serum ELISA positive results in the paratuberculosis-free flocks investigated in the present study. As a result, it seemed appropriate to use different specificity parameters for infected and uninfected farms.

Applying a test with an imperfect specificity at the individual level (ISp) to numerous individuals leads to very low specificity at the flock level (FSp), because FSp = ISp^n^, with n the number of animals tested [31,46]. Similar results would be obtained for serum pools, as shown by the results of the simulations we performed. Therefore, to our point of view, the use of pooled serum ELISA in a large-scale screening plan, is not recommendable in the absence of confirmation analysis using more specific methods such as culture or faecal PCR [47]. These confirmatory analyzes, however, would increase the overall cost of these screening strategies.

According to our simulation results, the use of qPCR on faecal mixtures appears very promising. Even if the detection sensitivity for farms with a low prevalence of infection (between 1 and 5%) is far from perfect (39–75%) when only 50 animals are tested, it increases very rapidly when the number tested animals and / or the prevalence of infection increase. In addition, the analysis of pools of size 20 or 10 instead of pools of size 5 would reduce the analytical costs of screening strategies and would yield for similar or slightly lower performances when infection prevalence is greater of equal to 10%. The only advantage of 5-fecal pools analyze would be the opportunity of getting a more refined, albeit still crude, assessment of the prevalence of infection. Finally, the proportion of highly contaminated (HC) individual fecal samples was low in our study (5.7% of qPCR-positive samples). Such a low prevalence of high-shedding animals might reduce the flock-sensitivity of screening strategies based on pool fecal qPCR when the infection prevalence is low, as those animals are the only one that can be invariably detected using pooled fecal analysis. Proportion of high-shedding animals ranging from 20% to 30% have be used in simulation studies by other authors [24,46], which might led to higher flock-sentivity estimates.

Nevertheless, establishing a Johne's Disease free status with a high confidence level (> 95%), based on the analysis of fecal pools would require the sampling of a very large number of animals (all the animals present for a 300 sheep flock). The costs of analysis to be incurred can therefore appear prohibitive. However, the accumulation of negative results in the context of a longitudinal monitoring of smaller scale (50 to 100 animals tested per year) but carried out over several years (3 to 6), could allow to achieve this goal, if the actual status of the flock does not change.

## Supporting information

S1 Fig ELISA work flow: Schematic representation of the simulation study approach for pooled serum ELISA. ISe_ELISA_: individual sensitivity; ISp_ELISA_: individual specificity; PSe_ELISA_: pooled-sample relative sensitivity for ELISA; PRSp_ELISA:_ pooled-sample relative specificity for ELISA; LP: lowly positive serum: sample (45% < S/P < 90%); HP: highly positive serum sample (S/P ≥ 90%).
(TIF)

S2 Fig PCR all: Number of qPCR-positive fecal pools detected, according to number and size of tested pools and simulated infection prevalence (all animals sampled, pool size 5 and 10). A: 30 pools of size 10; B: 60 pools of size 5. For a given simulated infection prevalence, figures in cells give the mean proportion of flocks that yield a given number of qPCR-positive fecal pools.
(TIF)

S3 Fig Pools size 20: Number of qPCR-positive fecal pools detected, according to number of tested pools and simulated infection prevalence (pools of size 20). A: 5 pools of size 20; B: 15 pools of size 20. For a given simulated infection prevalence, figures in cells give the mean proportion of flocks that yield a given number of qPCR-positive fecal pools.
(TIF)

S1 Table **Simulation model**: Assumptions and input parameters used for the simulation study aiming at estimating the flock sensitivity and specificity of screening strategies based on pooled fecal or pooled serum testing. (DOCX)

S2 Table Flock level distribution: Flock level distribution of serum ELISA S/P values and fecal qPCR Ct in 14 sheep flocks infected with paratuberculosis and in 3 paratuberculosis free flocks, France. (DOCX)
