# Supplementary figures and images for: Flock sensitivity and specificity of pooled fecal qPCR and pooled serum ELISA for screening ovine paratuberculosis
Source: PLoS One. 2019 Dec 26;14(12):e0226246. doi: 10.1371/journal.pone.0226246 (PMC6932769; doi:10.1371/journal.pone.0226246)

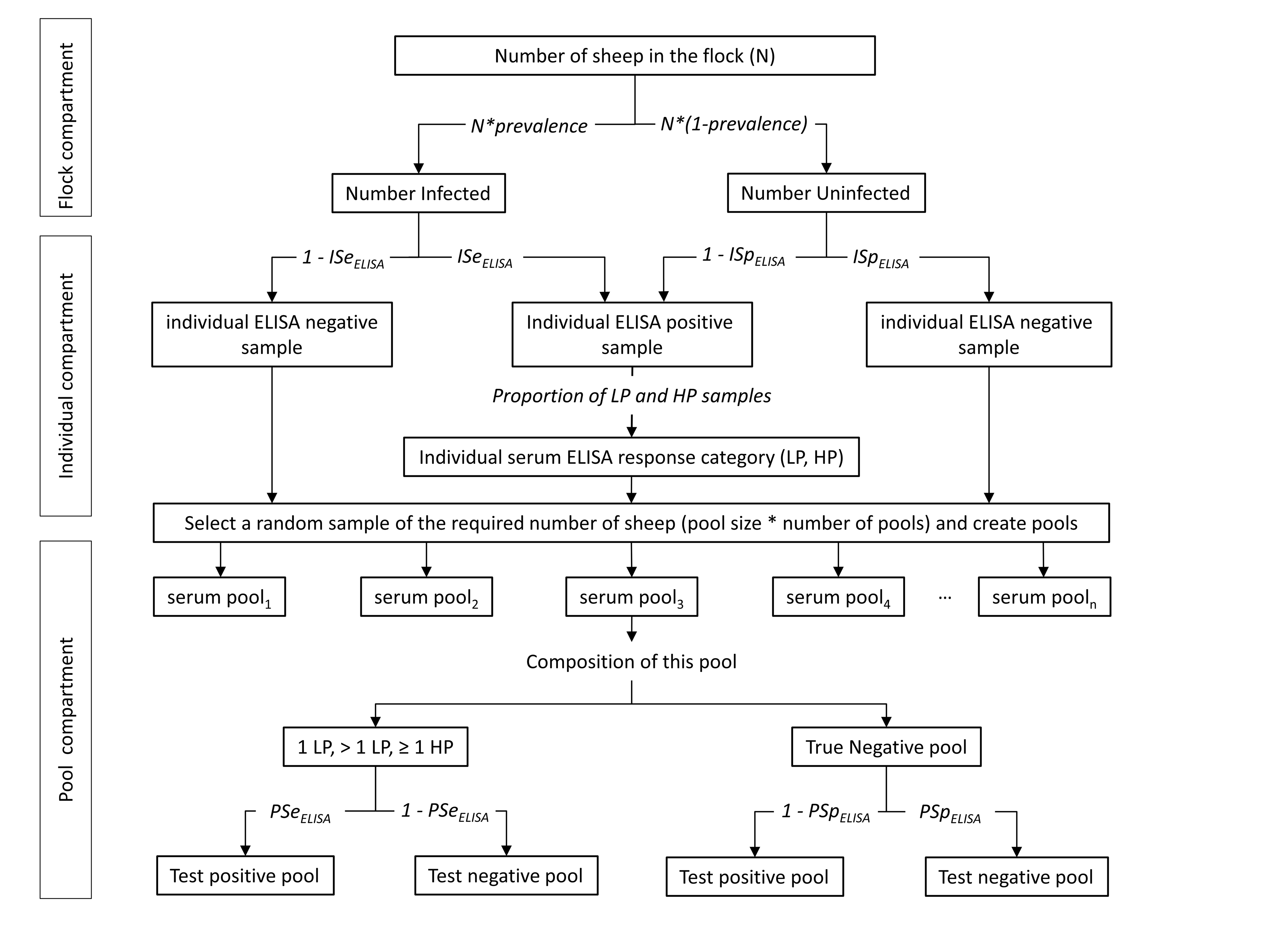

Supplement: S1 Fig — ISeELISA: individual sensitivity; ISpELISA: individual specificity; PSeELISA: pooled-sample relative sensitivity for ELISA; PRSpELISA: pooled-sample relative specificity for ELISA; LP: lowly positive serum: sample (45% < S/P < 90%); HP: highly positive serum sample (S/P ≥ 90%). (TIF) [file pone.0226246.s001.tif]

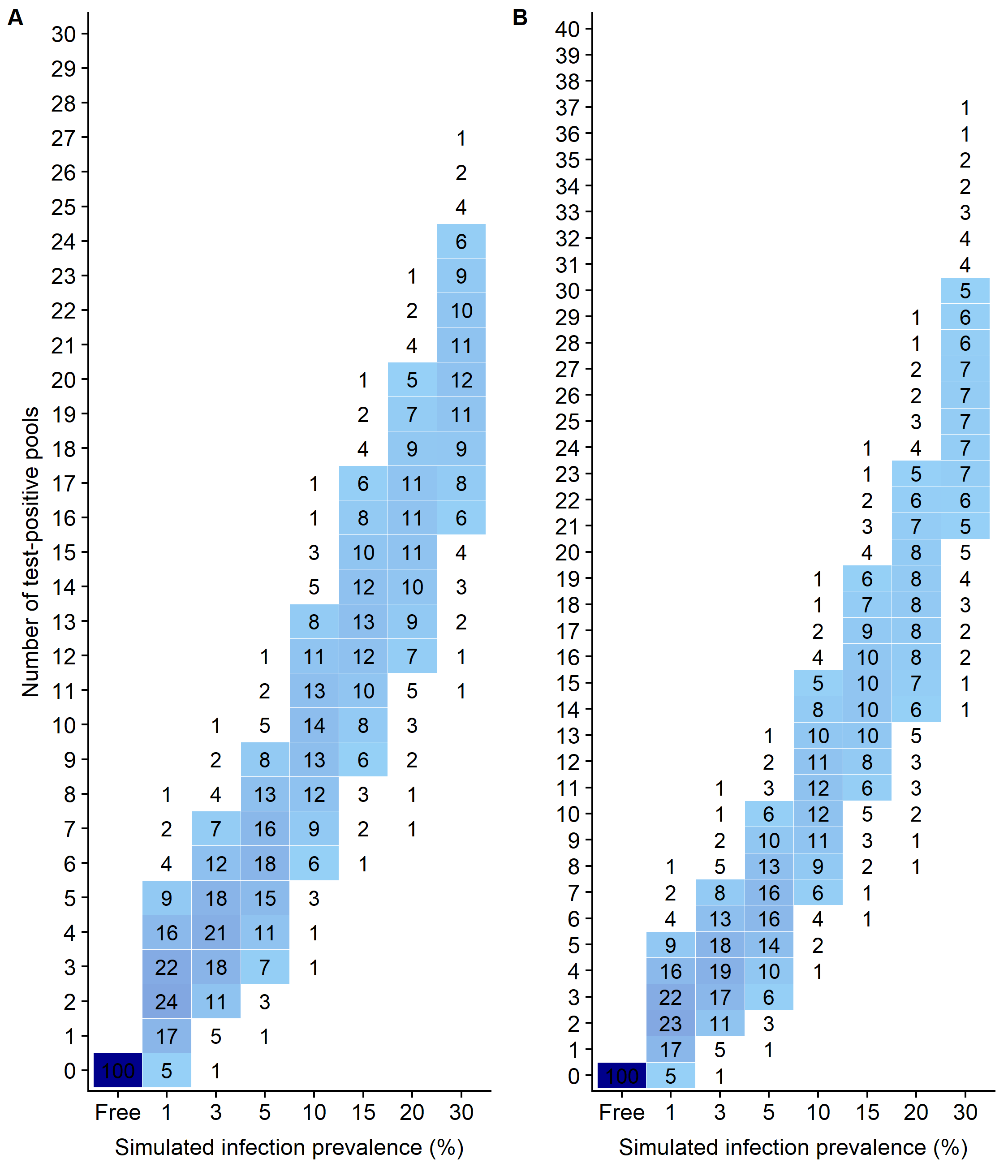

Supplement: S2 Fig — A: 30 pools of size 10; B: 60 pools of size 5. For a given simulated infection prevalence, figures in cells give the mean proportion of flocks that yield a given number of qPCR-positive fecal pools. (TIF) [file pone.0226246.s002.tif]

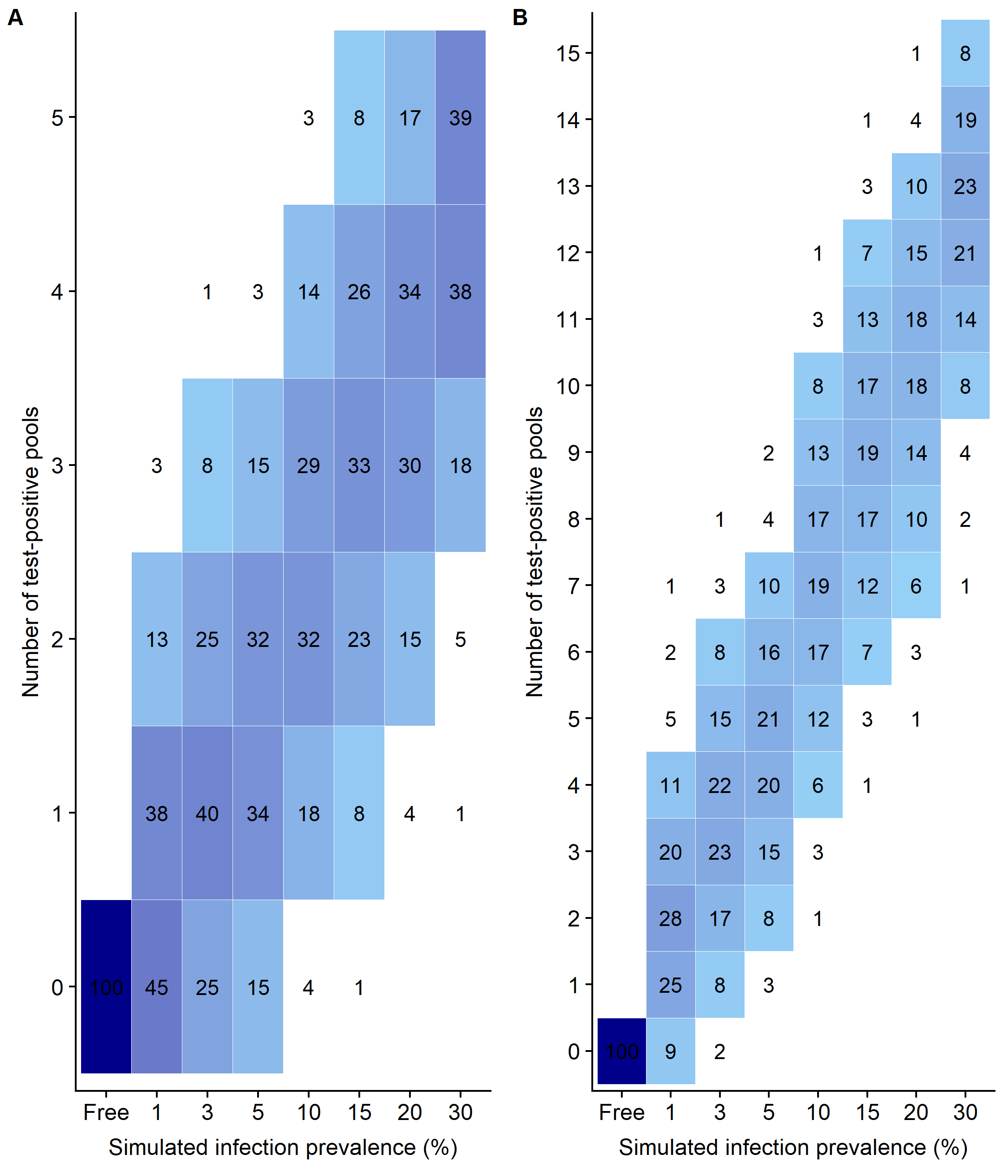

Supplement: S3 Fig — A: 5 pools of size 20; B: 15 pools of size 20. For a given simulated infection prevalence, figures in cells give the mean proportion of flocks that yield a given number of qPCR-positive fecal pools. (TIF) [file pone.0226246.s003.tif]
