# Supplementary material for: Flock sensitivity and specificity of pooled fecal qPCR and pooled serum ELISA for screening ovine paratuberculosis
Source: PLoS One. 2019 Dec 26;14(12):e0226246. doi: 10.1371/journal.pone.0226246 (PMC6932769; doi:10.1371/journal.pone.0226246)
Supplement: S1 Table — (DOCX) [file pone.0226246.s004.docx]

S2 Table - Supplementary materials for “Flock sensitivity and specificity of pooled fecal qPCR and pooled serum ELISA for screening ovine paratuberculosis”. Yoann Mathevon^1^, Gilles Foucras, Fabien Corbiere

**Simulation model: assumptions and input parameters.**

**Input values for parameter distributions**

Binomial or beta probability distributions were used to account for stochasticity and uncertainty about input parameters. For the pooled-sample relative sensitivity the number of pools built with at least one test-positive individual sample, and the number of positive pool results were used to construct α and β parameters of beta probability distributions, with α = number of positive pool results +1 and β = number of pools tested - number of positive pool results + 1. The same approach was used for pooled-sample specificities, based on the numbers of pools built with only test-negative individual samples and observed negative pool results. These distributions closely reflect the probability density functions of expected values under a binomial likelihood [1].

For simplification, some results from the experimental study were merged when possible.

For pooled serum ELISA, at the >25.0% decision thresholds for pools of size 5 and >15.0% for pools of size 10, no difference was evidenced between pools containing either only NL or up to 3 NH individual serums and results were gathered together. Similarly, results from serum pools constructed with one PH or 2 PL individual sample(s) were merged, as detection rates were the same. Finally, performances were similar for pools of size 5 and 10 (at the >25.0% and >15.0% decision thresholds) and the same beta distributions were therefore used for both pool sizes.

For the same reasons, results from pooled fecal qPCR based on an individual fecal amount of 3 or 10 grams were merged.

Although a limited number of true-negative fecal pools were tested, all yielded a negative result, and therefore the specificity of pooled fecal qPCR (PSp_qPCR_) was assumed to be perfect. The possibility of cross-contamination was assumed to be minimal due to inclusion of negative controls and other quality control procedures.

In the absence of experimental results the relative sensitivity for fecal pools containing *n* positive individual samples was modelled by PSe_qPCR_(*n*) = 1- (1- PSe_qPCR(1)_)*^n^*, where PSe_qPCR(1)_ is the detection rate for pools with one positive sample. This modelling approach is in strong agreement with recent results in cattle using fecal culture [2].

Input parameters, and distributions that were used to model these parameters are provided in the following tables 1 and 2.

# References

1. Messam LLM, Branscum AJ, Collins MT, Gardner IA. Frequentist and Bayesian approaches to prevalence estimation using examples from Johne’s disease. Animal Health Research Reviews. 2008;9:1‑23.

2. McKenna SLB, Ritter C, Dohoo I, Keefe GP, Barkema HW. Comparison of fecal pooling strategies for detection of Mycobacterium avium ssp. paratuberculosis in cattle. Journal of Dairy Science. 2018;101:7463‑70.

3. Mathevon Y, Foucras G, Falguières R, Corbiere F. Estimation of the sensitivity and specificity of two serum ELISAs and one fecal qPCR for diagnosis of paratuberculosis in sub-clinically infected young-adult French sheep using latent class Bayesian modeling. BMC Veterinary Research. 2017;13:230.

**Supplementary Table 1. Input parameters used for the flock and individual compartments of the simulation study.**

| **Parameter** | **Values** | **95% Confidence Interval** | **Distribution** |
| --- | --- | --- | --- |
| Flock size | 300 |  |  |
| Infection prevalence (%) | 0, 1, 3, 5, 10, 15, 20, 30 |  |  |
| Number of sheep sampled per flock | 50, 100, 300 |  |  |
| Pool size | 5, 10, 20 |  |  |
| Individual serum ELISA  ISe_ELISA_ (%), infected flock*  ISp_ELISA_ (%), infected flock*  ISp_ELISA_ (%), uninfected flock | 17.4  94.0  99.0 | 10.8 – 26.6  91.8 – 95.6  96.6 – 99.7 | Beta(16.3; 73.7)  Beta(583.8; 38.2)  Beta(210.0; 3.0) |
| Individual fecal qPCR  ISe_qPCR_ (%), infected flock*  ISp_qPCR_ (%), infected flock*  ISp_qPCR_ (%), uninfected flock | 47.5  99.0  100.0 | 26.2 – 69.9  97.6 – 99.6  - | Beta(8.8; 9.7)  Beta(487.4; 5.9)  - |
| % of Positive Low (PL) serum samples  Infected flock  Uninfected flock | 86.0  100.0 | 77.5 – 91.6  - | Beta(81.0; 14.0)  - |
| % of Low Contaminated (LC) fecal samples  Infected flock  Uninfected flock | 94.3  100.0 | 88.1 – 97.3  - | Beta(100.0; 7.0) |

* Derived from Mathevon et al (2017) [3]

ISe_ELISA_ and ISe_qPCR_: diagnostic sensitivity for serum ELISA and fecal qPCR at the individual level; ISp_ELISA_ and ISp_qPCR_: diagnostic specificity for serum ELISA and fecal qPCR at the individual level; Positive Low individual (PL) serum sample: serum sample with 45.0% < S/P < 90.0% when tested with IDEXX paratuberculosis screening kit (batch 5074, IDEXX, Montpellier, France); LC: Lowly Contaminated (LC) fecal sample: fecal sample with Ct ≥ 30 when tested with Adiavet ParaTB Real Time (BioX, Rochefort, Belgium)

**Supplementary Table 2. Input parameters used for the pool compartment of the simulation study**

| **Parameter** | **Values** | **95% Confidence Interval** | **Distribution** |
| --- | --- | --- | --- |
| Pooled serum ELISA  PRSe_ELISA_ (%), 1 PL  PRSe_ELISA_ (%), ≥ 2 PL or ≥ 1 PH  PRSp_ELISA_ (%) | 62.2  100.0  100.0 | 46.0 – 76.0  91.8 – 99.9  96.5 – 100.0 | Beta(24.0; 15.0)  Beta(43.0, 1.0)  Beta(103.0, 1.0) |
| Pooled fecal qPCR  PRSe_qPCR_ (%), 1 LC, pool size 5  PRSe_qPCR_ (%), 1 LC, pool size 10  PRSe_qPCR_ (%), 1 LC, pool size 20  PRSe_qPCR_ (%), 1 HC, pool size 5  PRSe_qPCR_ (%), 1 HC, pool size 10  PRSe_qPCR_ (%), 1 HC, pool size 20  PRSp_qPCR_ (%) | 89.0  68.2  53.0  99.2  100.0  100.0  100.0 | 79.8 – 94.3  57.7 – 77.1  36.0 – 69.8  95.5 – 99.8  97.1 – 100.0  97.0 – 100.0  - | Beta(66.0; 9.0)  Beta(59.0; 28.0)  Beta(17.0; 15.0)  Beta(122.0; 2.0)  Beta(126.0; 1.0)  Beta(121.0; 1.0)  - |

PRSe_ELISA_  and PRSe_qPCR_: pool relative sensitivity for ELISA and qPCR, defined as the proportion of pools containing at least one test-positive individual sample that will yield a positive result, i.e. Pr(pool test +| ≥ 1 test-positive individual sample); PRSp_ELISA_ and PRSp_qPCR_: pool relative specificity for ELISA and qPCR, defined as the proportion of pools containing only test-negative individual samples that yielded a negative result, i.e. Pr(pool test – | only test negative individual samples) ; PL: Positive Low individual serum sample: serum sample with 45.0% < S/P < 90.0% when tested with IDEXX paratuberculosis screening kit (batch 5074, IDEXX, Montpellier, France); PH: Positive High individual serum sample: serum sample with S/P ≥90.0%; LC: Lowly Contaminated fecal sample : fecal sample with Ct ≥ 30 when tested with Adiavet ParaTB Real Time (BioX, Rochefort, Belgium); HC: Highly Contaminated fecal sample : fecal sample with Ct < 30 .
