# Supplementary material for: Flock sensitivity and specificity of pooled fecal qPCR and pooled serum ELISA for screening ovine paratuberculosis
Source: PLoS One. 2019 Dec 26;14(12):e0226246. doi: 10.1371/journal.pone.0226246 (PMC6932769; doi:10.1371/journal.pone.0226246)
Supplement: S2 Table — (DOCX) [file pone.0226246.s005.docx]

S3 table: Supplementary materials for “Flock sensitivity and specificity of pooled fecal qPCR and pooled serum ELISA for screening ovine paratuberculosis”. Yoann Mathevon^1^, Gilles Foucras, Fabien Corbiere

**flock level distribution of serum ELISA S/P values and fecal qPCR Ct in 14 sheep flocks infected with paratuberculosis and in 3 paratuberculosis free flocks, France.**

| **Flock** | **# sampled**  **sheep** |  | | **Serum ELISA S/P value (%)** | | | |  | | **Fecal qPCR Ct** | | | |  |
| --- | --- | --- | --- | --- | --- | --- | --- | --- | --- | --- | --- | --- | --- | --- |
|  |  |  | < 22.5  (NL) | | [22.5 – 45.0[  (NH) | [45.0 – 90.0[  (PL) | ≥ 90.0  PH | |  | | >42  (Neg) | [30-42]  (LC) | ≤ 30  (HC) | |
| **Infected flocks** | |  |  | |  |  |  | |  | |  |  |  | |
| A | 59 |  | 50 | | 5 | 3 | 1 | |  | | 53 | 5 | 1 | |
| B | 140 |  | 124 | | 11 | 5 | 0 | |  | | 129 | 11 | 0 | |
| C | 66 |  | 63 | | 2 | 1 | 0 | |  | | 64 | 2 | 0 | |
| D | 85 |  | 65 | | 6 | 12 | 2 | |  | | 64 | 19 | 2 | |
| E | 66 |  | 63 | | 2 | 1 | 0 | |  | | 64 | 2 | 0 | |
| F | 79 |  | 67 | | 6 | 6 | 0 | |  | | 56 | 23 | 0 | |
| G | 90 |  | 62 | | 15 | 11 | 2 | |  | | 77 | 12 | 1 | |
| H | 64 |  | 41 | | 15 | 8 | 0 | |  | | 62 | 2 | 0 | |
| I | 92 |  | 73 | | 4 | 11 | 4 | |  | | 91 | 1 | 0 | |
| J | 103 |  | 78 | | 13 | 10 | 2 | |  | | 99 | 3 | 1 | |
| K | 95 |  | 88 | | 6 | 1 | 0 | |  | | 95 | 0 | 0 | |
| L | 100 |  | 87 | | 10 | 2 | 1 | |  | | 100 | 0 | 0 | |
| M | 77 |  | 66 | | 5 | 5 | 1 | |  | | 67 | 10 | 0 | |
| N | 81 |  | 71 | | 6 | 4 | 0 | |  | | 71 | 9 | 1 | |
| **Total** | **1197** |  | **998** | | **106** | **80** | **13** | |  | | **1092** | **99** | **6** | |
|  |  |  |  | |  |  |  | |  | |  |  |  | |
|  | |  |  | |  |  |  | |  | |  |  |  | |
|  | |  |  | |  |  |  | |  | |  |  |  | |
|  | |  |  | |  |  |  | |  | |  |  |  | |
| **Flock** | **# sampled**  **sheep** |  | | **Serum ELISA S/P value (%)** | | | |  | | **Fecal qPCR Ct** | | | |  |
|  |  |  | < 22.5  (NL) | | [22.5 – 45.0[  (NH) | [45.0 – 90.0[  (PL) | ≥ 90.0  PH | |  | | >42  (Neg) | [30-42]  (LC) | ≤ 30  (HC) | |
| **Uninfected flocks** | |  |  | |  |  |  | |  | |  |  |  | |
| O | 125* |  | 104 | | 20 | 1 | 0 | |  | | 125 | 0 | 0 | |
| P | 91** |  | 87 | | 3 | 1 | 0 | |  | | 91 | 0 | 0 | |
| Q | 171 |  | ND | | ND | ND | ND | |  | | 171 | 0 | 0 | |
| **Total** | **387** |  | **191** | | **23** | **2** | **0** | |  | | **387** | **0** | **0** | |

* 3 sheep with missing serum ELISA result; ** 2 sheep with missing serum ELISA result
